# Supplementary material for: Investigating the potential of X chromosome shredding for mouse genetic biocontrol
Source: Sci Rep. 2024 Jun 12;14:13466. doi: 10.1038/s41598-024-63706-4 (PMC11169450; doi:10.1038/s41598-024-63706-4)
Supplement: Supplementary file 1 — Supplementary Information. [file 41598_2024_63706_MOESM1_ESM.pdf]

Investigating the potential of X chromosome shredding for mouse genetic biocontrol

Mark D. Bunting, Gelshan I. Godahewa, Nicole O. McPherson, Louise J. Robertson, Luke Gierus, Sandra G. Piltz, Owain Edwards, Mark Tizard and Paul Q. Thomas

Supplementary Figures and Tables

Supplementary figure 1

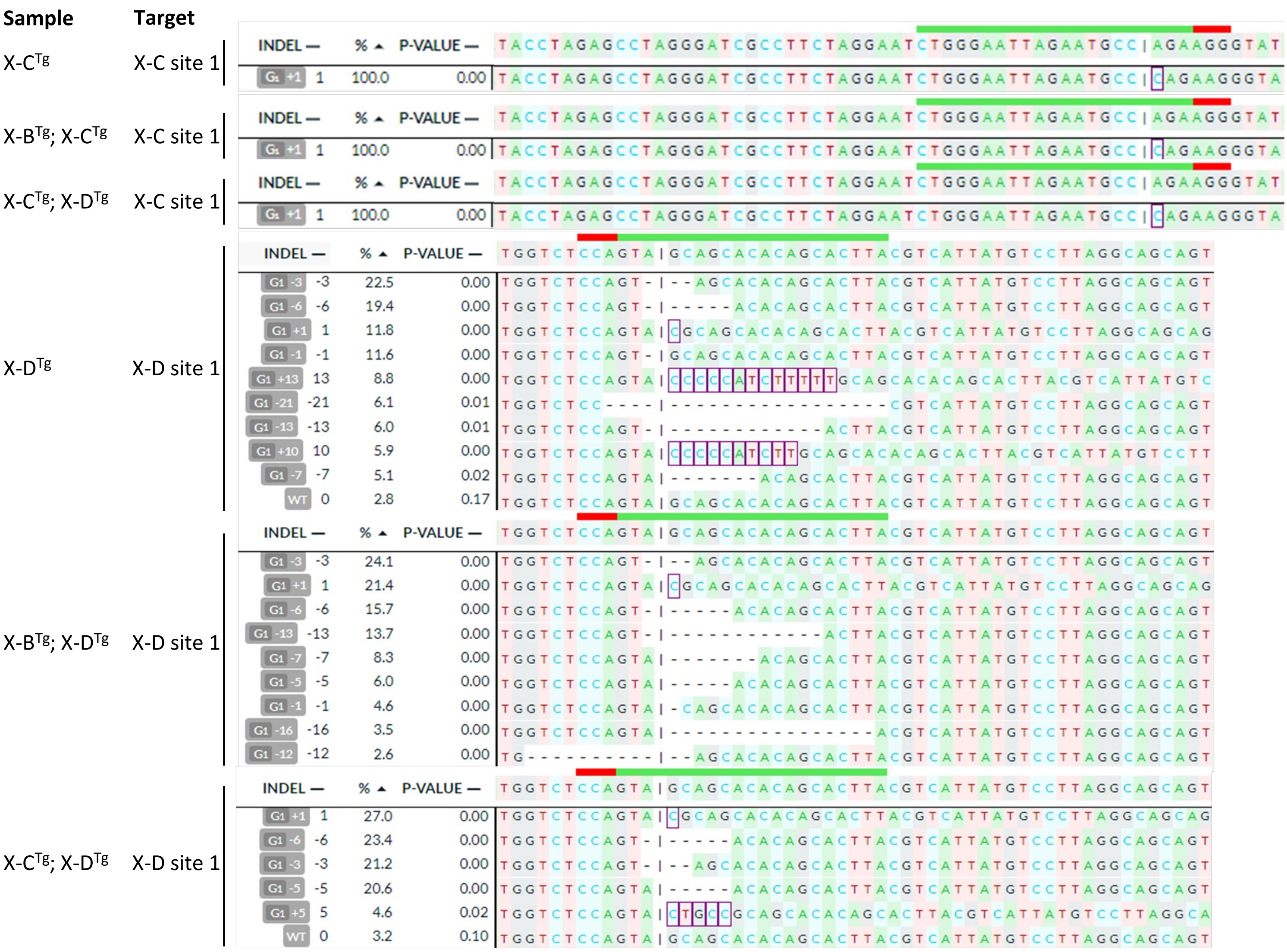

Supplementary Figure 1. Molecular evidence of X chromosome-targeted Cas9 activity in mouse ES cells. Genomic DNA from the surviving cells was isolated and a single X-C gRNA target site and X-D gRNA target site was amplified and sequenced. The presence of indels is shown in either single gRNA or dual gRNA plasmid transfected cells.

Supplementary figure 2

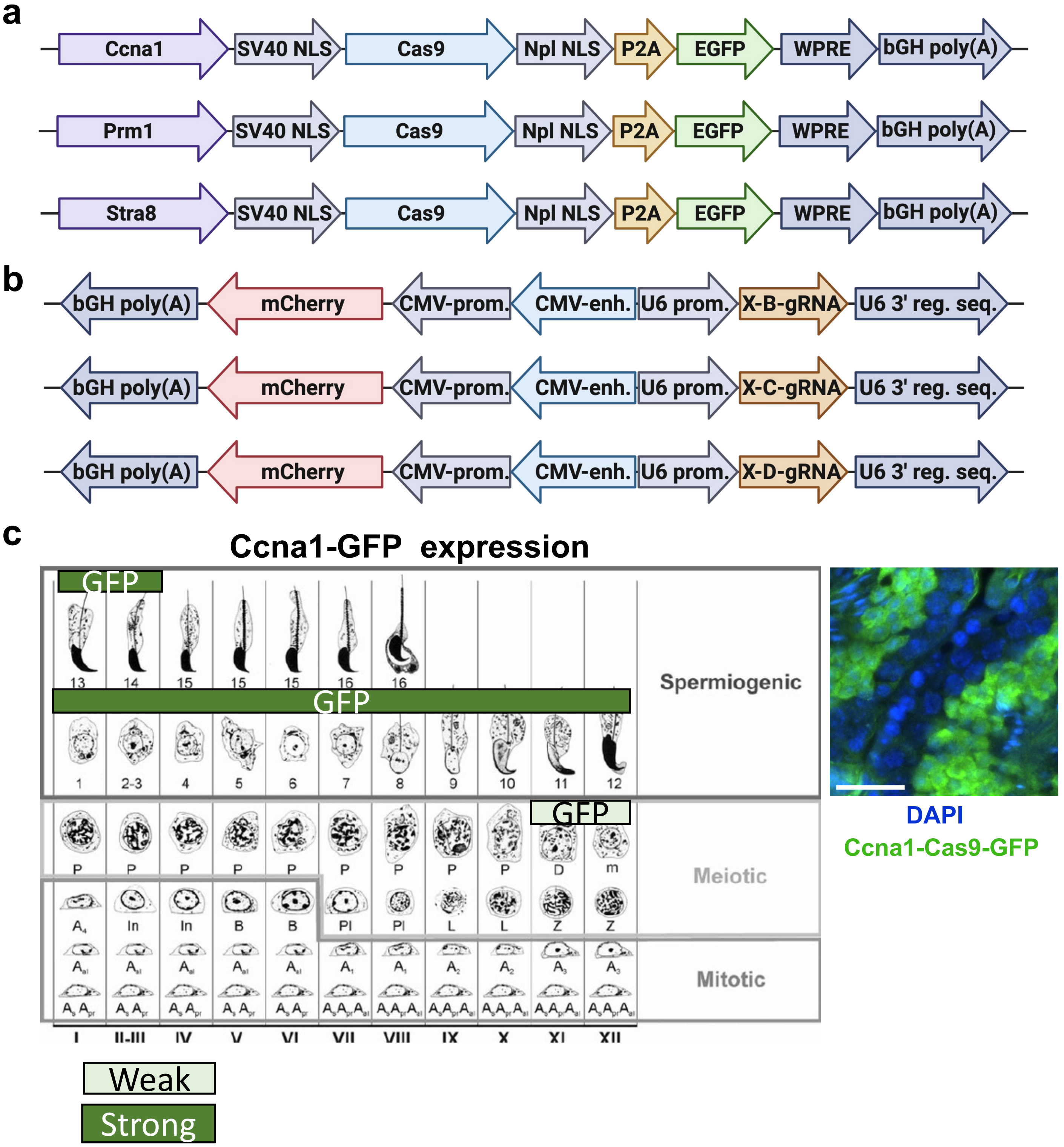

Supplementary Figure 2. Transgene construct design. (**a**) Murine germline-specific promoters *Ccna1*, *Prm1*, and *Stra8* were inserted upstream of an NLS-flanked Cas9 coding sequence followed by the self-cleaving linker, P2A, EGFP, WPRE and a bGH poly(A). (**b**) Expression of X shredder gRNAs was driven by the human U6 promoter and supported by the U6 3' regulatory sequence downstream of the guide scaffold sequence. In the opposite direction, mCherry expression was driven by CMV enhancer and CMV promoter sequence. (**c**) Schematic of sperm development stage with *Ccna1*-Cas9-EGFP construct expression indicated as weak (light green) and strong (dark green) boxes assessed from multiple fields of view of multiple mice. Adapted from Russell LD, Ettlin RA, Sinha Hikim AP, Clegg ED (1990) Histological and histopathological evaluation of the testis. Clearwater: Cache River Press. Representative IF of Cas9-GFP expression (green) in the testis of Ccna1-Cas9-GFP transgenic mice merged with DAPI (blue). Scale bar = 30 μM.

Supplementary figure 3

a

Blastocysts from Ccna1^{Tg}; X-D^{Tg} male x WT female

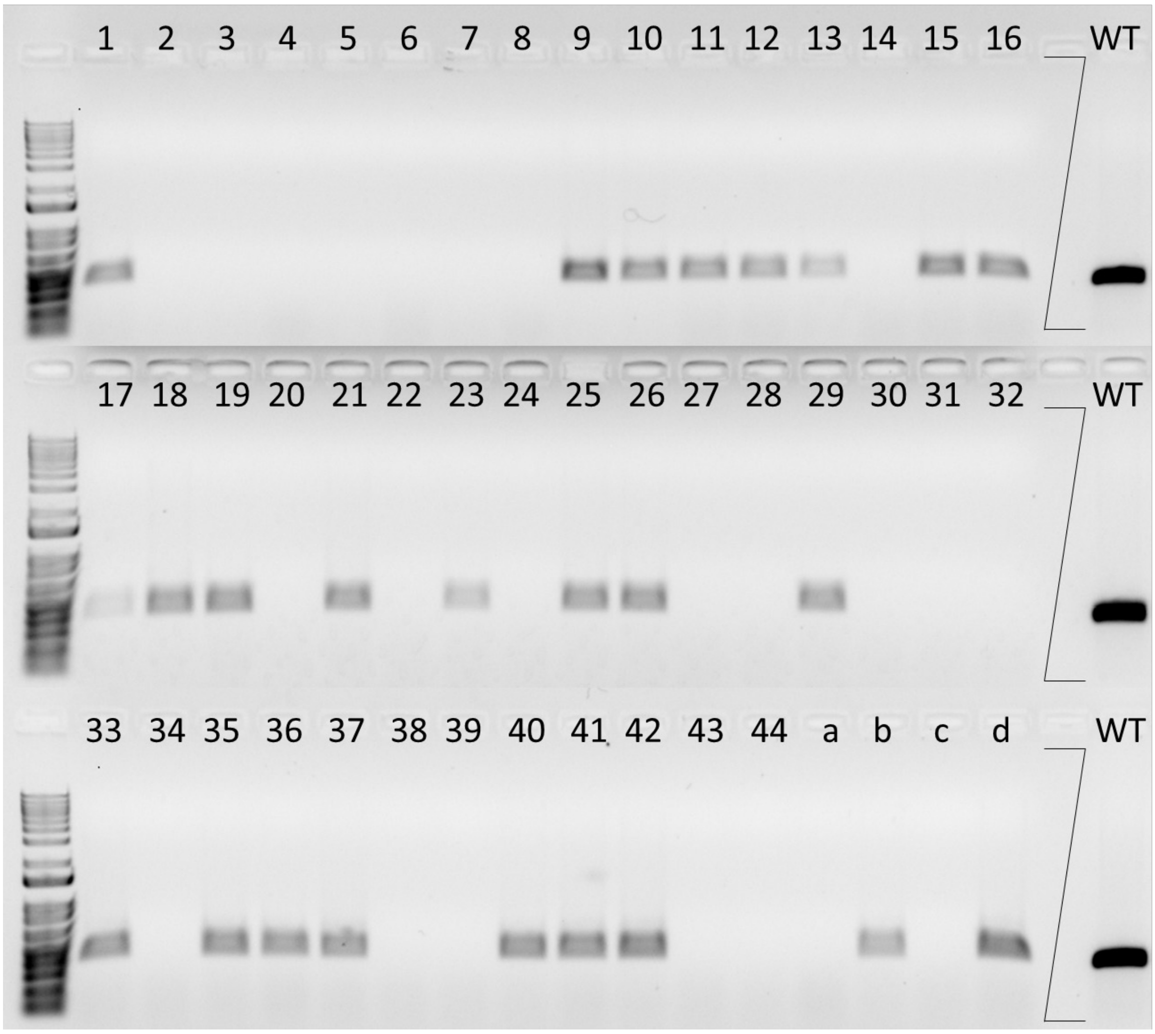

b

Ccna1^{Tg}; X-D^{Tg} blastocysts

Sample	Blastocyst No	Indel	Contribution
Ccna1^{Tg}; X-D^{Tg}	#41	0	100%
	#42	0	100%
	#43	0	100%
	#44	0	100%
	a	0	100%
	b	0	100%

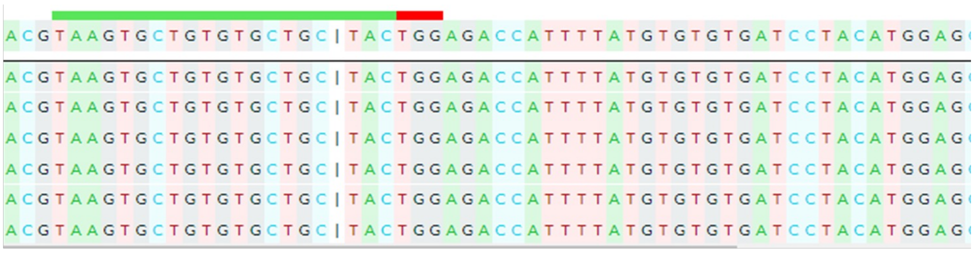

Supplementary Figure 3. Sex of 44 blastocysts (23 male, 21 female) from *in vitro* fertilisation using sperm from *Ccna1*^{Tg}; X-D^{Tg} males. (**a**) PCR for the Y-chromosomal *Sry* gene in DNA extracted from blastocysts generated using *Ccna1*^{Tg}; X-D^{Tg} sperm and WT oocytes. Blastocysts b and d are male and blastocysts a and c are female controls. (**b**) Sanger sequencing of a single X-D gRNA target site on the X chromosome.

Supplementary figure 4

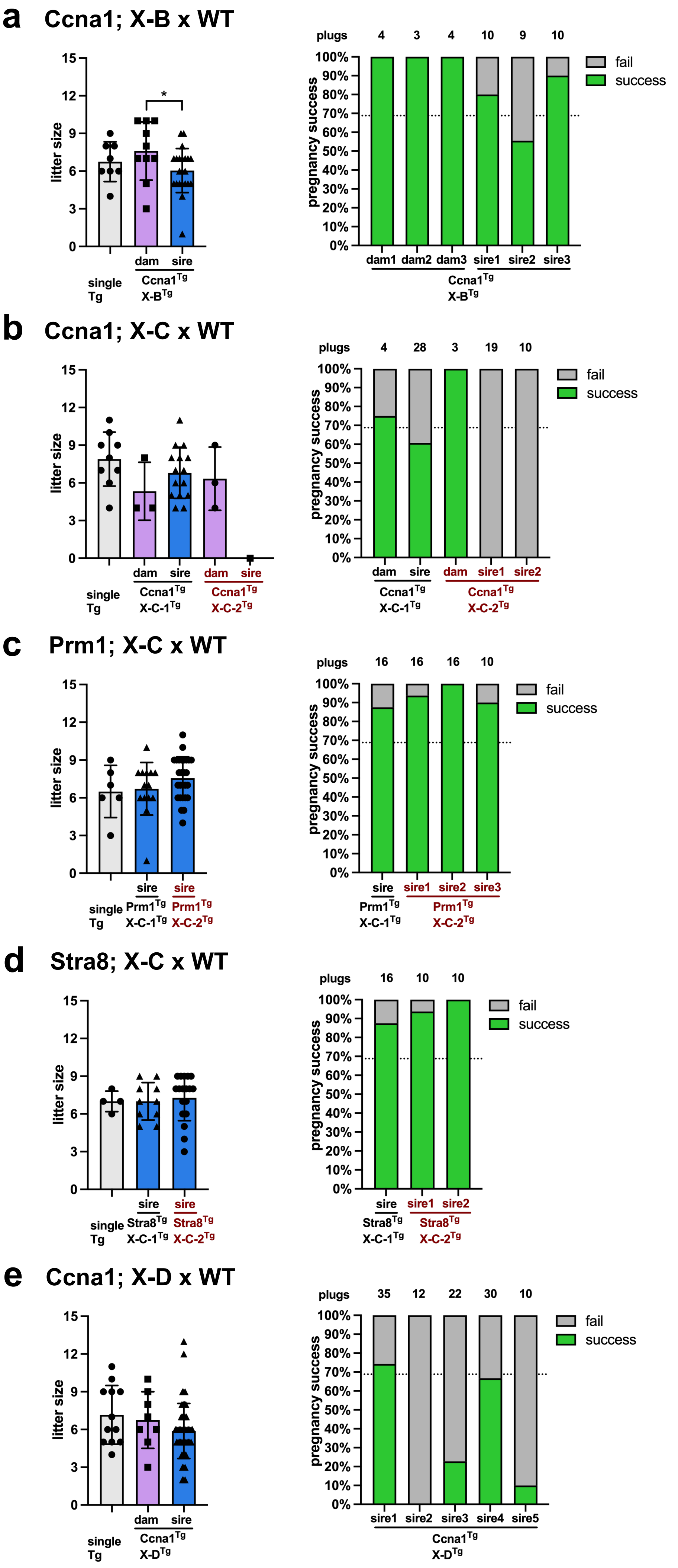

Supplementary Figure 4. Fertility impacts of X shredder activity. Litter size (left) and pregnancy success as determined by plug and successful pregnancy counts (right) were performed. (**a**) *Ccna1*^{Tg}; X-B^{Tg} x WT matings. Single Tg n = 8, *Ccna1*^{Tg}; X-B^{Tg} dam n = 10, *Ccna1*^{Tg}; X-B^{Tg} sire n = 22. (**b**) *Ccna1*^{Tg}; X-C^{Tg} x WT matings (two independent X-C^{Tg} founder lines). Single Tg n = 9, *Ccna1*^{Tg}; X-C-1^{Tg} dam n = 3, *Ccna1*^{Tg}; X-C-1^{Tg} sire n = 15, *Ccna1*^{Tg}; X-C-2^{Tg} dam n = 3, *Ccna1*^{Tg}; X-C-2^{Tg} sire n = 0. (**c**) *Prm1*^{Tg}; X-C^{Tg} x WT matings (two independent X-C^{Tg} founder lines). Single Tg n = 6, *Prm1*^{Tg}; X-C-1^{Tg} sire n = 14, *Prm1*^{Tg}; X-C-2^{Tg} sire n = 27. (**d**) *Stra8*^{Tg}; X-C^{Tg} x WT matings (two independent X-C^{Tg} founder lines). Single Tg n = 4, *Stra8*^{Tg}; X-C-1^{Tg} sire n = 10, *Stra8*^{Tg}; X-C-2^{Tg} sire n = 18. (**e**) *Ccna1*^{Tg}; X-D^{Tg} x WT matings. Single Tg n = 12, *Ccna1*^{Tg}; X-D^{Tg} dam n = 8, *Ccna1*^{Tg}; X-D^{Tg} sire n = 50.

Supplementary figure 5

a Sperm from sires - X chromosome indels

	Mouse ID	Indel	Contribution	Sequence
X-C^{Tg}	3.1.5	0	100%	CTAGGAATCTGGGAATTAGAATGCC ¦ AGAAGGGTATAGCGCATGATTTTTTTAGGGGGCGGGGCTGGAAGGGGGGG
	4.5.84	0	100%	CTAGGAATCTGGGAATTAGAATGCC ¦ AGAAGGGTATAGCGCATGATTTTTTAAGGGGGCGGGGTGGGAAGGGGGGG
Prm1^{Tg}; X-C^{Tg}	2.1d	0	100%	CTAGGAATCTGGGAATTAGAATGCC ¦ AGAAGGGTATAGCGCATGATTTTTTTAGGGGGCGGGGCTGGAAGGGGGGG
	2.1f	0	100%	CTAGGAATCTGGGAATTAGAATGCC ¦ AGAAGGGTATAGCGCATGATTTTTTTAGGGGGCGGGGCTGGAAGGGGGGG
	2.1g	0	100%	CTAGGAATCTGGGAATTAGAATGCC ¦ AGAAGGGTATAGCGCATGATTTTTTTAGGGGGCGGGGCTGGAAGGGGGGG
	1.2e	0	100%	CTAGGAATCTGGGAATTAGAATGCC ¦ AGAAGGGTATAGCGCATGATTTTTTTAGGGGGCGGGGCTGGAAGGGGGGG
		-1	3%	CTAGGAATCTGGGAATTAGAATGCC ¦ -GAAGGGTATAGCGCATGATTTTTTTAGGGGGCGGGGCTGGAAGGGGGGG
		+1	1%	CTAGGAATCTGGGAATTAGAATGCC ¦ NAGAAGGGTATAGCGCATGATTTTTTTAGGGGGCGGGGCTGGAAGGGGGG
Stra8^{Tg}; X-C^{Tg}	1.1.9	0	100%	CTAGGAATCTGGGAATTAGAATGCC ¦ AGAAGGGTATAGCGCATGATTTTTTTAGGGGGCGGGGCTGGAAGGGGGGG
	2.1.23	0	100%	CTAGGAATCTGGGAATTAGAATGCC ¦ AGAAGGGTATAGCGCATGATTTTTTTAGGGGGCGGGGCTGGAAGGGGGGG
	2.1.24	0	100%	CTAGGAATCTGGGAATTAGAATGCC ¦ AGAAGGGTATAGCGCATGATTTTTTTAGGGGGCGGGGCTGGAAGGGGGGG

b Male experimental offspring - X chromosome indels

	Mouse ID	Indel	Contribution	Sequence
Ccna1^{Tg}; X-D^{Tg}	12.1.30	0	78%	TAAGTGCTGTGTGCTGC ¦ TACTGGAGACCATTTTATGTGTGTGATCCTACATGGAGCCTGAA
		-3	22%	TAAGTGCTGTGTGC--- ¦ TACTGGAGACCATTTTATGTGTGTGATCCTACATGGAGCCTGAA

c Female experimental offspring - no X chromosome indels

	Mouse ID	Indel	Contribution	Sequence
Prm1^{Tg}; X-C^{Tg}	5.1.12	0	100%	CTAGGAATCTGGGAATTAGAATGCC ¦ AGAAGGGTATAGCGCATGATTTTTTTAGGGGGCGGGGTTGG
	5.1.14	0	100%	CTAGGAATCTGGGAATTAGAATGCC ¦ AGAAGGGTATAGCGCATGATTTTTTTAGGGGGCGGGGTTGG
	5.2.19	0	100%	CTAGGAATCTGGGAATTAGAATGCC ¦ AGAAGGGTATAGCGCATGATTTTTTTAGGGGGCGGGGTTGG
	6.1.25	0	100%	CTAGGAATCTGGGAATTAGAATGCC ¦ AGAAGGGTATAGCGCATGATTTTTTTAGGGGGCGGGGTTGG
Stra8^{Tg}; X-C^{Tg}	3.2.40	0	100%	CTAGGAATCTGGGAATTAGAATGCC ¦ AGAAGGGTATAGCGCATGATTTTTTTAGGGGGCGGGGTTGG
	3.2.41	0	100%	CTAGGAATCTGGGAATTAGAATGCC ¦ AGAAGGGTATAGCGCATGATTTTTTTAGGGGGCGGGGTTGG
	3.2.42	0	100%	CTAGGAATCTGGGAATTAGAATGCC ¦ AGAAGGGTATAGCGCATGATTTTTTTAGGGGGCGGGGTTGG
	3.2.43	0	100%	CTAGGAATCTGGGAATTAGAATGCC ¦ AGAAGGGTATAGCGCATGATTTTTTTAGGGGGCGGGGTTGG
Ccna1^{Tg}; X-C^{Tg}	6.2.21	0	100%	CTAGGAATCTGGGAATTAGAATGCC ¦ AGAAGGGTATAGCGCATGATTTTTTTAGGGGGCGGGGTTGG
	6.2.22	0	100%	CTAGGAATCTGGGAATTAGAATGCC ¦ AGAAGGGTATAGCGCATGATTTTTTTAGGGGGCGGGGTTGG
	9.1.24	0	100%	CTAGGAATCTGGGAATTAGAATGCC ¦ AGAAGGGTATAGCGCATGATTTTTTTAGGGGGCGGGGTTGG
	9.1.25	0	100%	CTAGGAATCTGGGAATTAGAATGCC ¦ AGAAGGGTATAGCGCATGATTTTTTTAGGGGGCGGGGTTGG
Ccna1^{Tg}; X-D^{Tg}	14.1.53	0	100%	TAAGTGCTGTGTGCTGC ¦ TACTGGAGACCATTTTATGTGTGTGATCCTACATGGAGCCT
	14.1.54	0	100%	TAAGTGCTGTGTGCTGC ¦ TACTGGAGACCATTTTATGTGTGTGATCCTACATGGAGCCT
	14.1.55	0	100%	TAAGTGCTGTGTGCTGC ¦ TACTGGAGACCATTTTATGTGTGTGATCCTACATGGAGCCT
	18.1.87	0	100%	TAAGTGCTGTGTGCTGC ¦ TACTGGAGACCATTTTATGTGTGTGATCCTACATGGAGCCT

Supplementary Figure 5. Molecular characterisation of X shredder transgenic male sperm and X chromosome target sites in female offspring from experimental matings. (**a**) Sperm from ex-breeder X-C^{Tg}, *Prm1*^{Tg}; X-C^{Tg} and *Stra8*^{Tg}; X-C^{Tg} males was isolated and Sanger sequencing performed for the respective X chromosome target sites to identify the presence of indels. (**b**) Genomic DNA of a male pup from a *Ccna1*^{Tg}; X-D^{Tg} male x WT female mating was isolated and a single X-D gRNA target site was sequenced. The presence of a small proportion of indels is shown. (**c**) Genomic DNA of female pups from a *Prm1*^{Tg}; X-C^{Tg} male x WT female, *Stra8*^{Tg}; X-C^{Tg} male x WT female, *Ccna1*^{Tg}; X-C^{Tg} male x WT female, and *Ccna1*^{Tg}; X-D^{Tg} male x WT female mating was isolated and a single X-C gRNA target site was sequenced. No indels were detected in any of the analysed female offspring.

Supplementary table 1 – gRNA used in this study

sgRNA	Sequence
X-B	GCTTGGTTAGGGTGAGTGCT
X-C	CTGGGAATTAGAATGCCAGA
X-D	TAAGTGCTGTGTGCTGCTAC
Tyrosinase	TCAGTTCCCCTTCAAAGGGG
Neomycin	GGCAGCGCGGCTATCGTGGC
mCherry	GGCCACGAGTTCGAGATCGA

Supplementary table 2 – oligonucleotides used in this study

**Oligo**	**Sequence**
X-B Forward	CACCGCTTGGTTAGGGTGAGTGCT
X-B Reverse	AAACAGCACTCACCCTAACCAAGC
X-C Forward	CACCGCTGGGAATTAGAATGCCAGA
X-C Reverse	AAACTCTGGCATTCTAATTCCCAGC
X-D Forward	CACCGTAAGTGCTGTGTGCTGCTAC
X-D Reverse	AAACGTAGCAGCACACAGCACTTAC
Sequencing oligo 1	AATTTCTTGGGTAGTTTGCAGT
Sequencing oligo 2	GGTTTCGCCACCTCTGACTTG
Ccna1-Cas9-EGFP genotyping Forward	GGCACTCCAGAGTTCTCTGC
Ccna1-Cas9-EGFP genotyping Reverse	CTTGTACTCGTCGGTGATCAC
X shredder genotyping common Forward	ACTGCCAAGTAGGAAAGTCCCA
X-B genotyping Reverse	AAACAGCACTCACCCTAACCAAGC
X-C genotyping Reverse	AAACTCTGGCATTCTAATTCCCAGC
X-D genotyping Reverse	AAACGTAGCAGCACACAGCACTTAC
X-D single cut site Forward	GGTTCTCAAGCACCAGCACAGATC
X-D single cut site Reverse	AGGAGTTCCAGTTTGTCTGTGG
X-C single cut site Forward	GAGGTGTGGAGAATTCACTAGAGGTCTC
X-C single cut site Reverse	GGAAGCAGCGGCAGGATTAA
X-B Large Deletion Forward	GAATTGAACCACAACCACGGTC
X-B Large Deletion Reverse	GGAGAGAAGGTGGAGTGGATAAG
Rpgr qPCR Forward	GATGACGATGAAGTGGAGACTGAGA
Rpgr qPCR Reverse	GCCTGTACTACTTGCTCCACTACT
TLR7 qPCR Forward	TGGAGAGCCGGTGATAACAGATAC
TLR7 qPCR Reverse	TCAGTAACTGGAGTCTGTCCCAAA
DMD qPCR Forward	CTGTTGGAGGTACCTGCACTGGC
DMD qPCR Reverse	ACTCGATCAAGCAGAGACAGCCA
